# Pairwise genetic meta-analyses between schizophrenia and substance dependence phenotypes reveals novel association signals with pharmacological significance

**DOI:** 10.1038/s41398-022-02186-4

**Published:** 2022-09-23

**Authors:** Laura A. Greco, William R. Reay, Christopher V. Dayas, Murray J. Cairns

**Affiliations:** 1 School of Biomedical Sciences and Pharmacy, The University of Newcastle, Callaghan, NSW Australia; 2 Precision Medicine Research Program, Hunter Medical Research Institute, New Lambton, NSW Australia

**Keywords:** Schizophrenia, Personalized medicine

## Abstract

Almost half of individuals diagnosed with schizophrenia also present with a substance use disorder, however, little is known about potential molecular mechanisms underlying this comorbidity. We used genetic analyses to enhance our understanding of the molecular overlap between these conditions. Our analyses revealed a positive genetic correlation between schizophrenia and the following dependence phenotypes: alcohol (*r*_*g*_ = 0.368, SE = 0.076, *P* = 1.61 × 10^−6^), cannabis use disorder (*r*_*g*_ = 0.309, SE = 0.033, *P* = 1.97 × 10^−20^) and nicotine (*r*_*g*_ = 0.117, SE = 0.043, *P* = 7.0 × 10^−3^), as well as drinks per week (*r*_*g*_ = 0.087, SE = 0.021, *P* = 6.36 × 10^−5^), cigarettes per day (*r*_*g*_ = 0.11, SE = 0.024, *P* = 4.93 × 10^−6^) and life-time cannabis use (*r*_*g*_ = 0.234, SE = 0.029, *P* = 3.74 × 10^−15^). We further constructed latent causal variable (LCV) models to test for partial genetic causality and found evidence for a potential causal relationship between alcohol dependence and schizophrenia (GCP = 0.6, SE = 0.22, *P* = 1.6 × 10^−3^). This putative causal effect with schizophrenia was not seen using a continuous phenotype of drinks consumed per week, suggesting that distinct molecular mechanisms underlying dependence are involved in the relationship between alcohol and schizophrenia. To localise the specific genetic overlap between schizophrenia and substance use disorders (SUDs), we conducted a gene-based and gene-set pairwise meta-analysis between schizophrenia and each of the four individual substance dependence phenotypes in up to 790,806 individuals. These bivariate meta-analyses identified 44 associations not observed in the individual GWAS, including five shared genes that play a key role in early central nervous system development. The results from this study further supports the existence of underlying shared biology that drives the overlap in substance dependence in schizophrenia, including specific biological systems related to metabolism and neuronal function.

## Introduction

Schizophrenia is a complex and debilitating psychiatric disorder with a diverse array of symptoms and comorbidities including substance dependence [1]. While the onset generally occurs during early adolescence, schizophrenia is highly heritable, which provides an opportunity to glean molecular insight into the composition of dysfunctional networks underlying its pathophysiology and phenotypic diversity, including associated comorbidities. Substance use disorders (SUDs) are also complex, heritable traits with susceptibility linked to unique polygenic architecture not shared with non-pathological substance use behaviour [2]. Twin studies have provided insights into the magnitude of total SUDs heritability regardless of the substance [3]; alcohol (AD, 0.63), cannabis (CD, 0.78) nicotine (ND, 0.72), cocaine (CoD, 0.61), and opioids (OD, 0.61) [4].

While these SUDs arise independently of psychotic and affective disorders, they are frequently comorbid. A 2018 meta-analysis by Hunt et al. of 123 articles between 1990 and 2017 (*n* = 165,811) found that the prevalence of any SUD in schizophrenia was 41.7%, with 26.2% for cannabis, 24.3% for alcohol and 7.3% for stimulants [5]. Some population studies have reported even higher use, with 72% of people with schizophrenia being daily cigarette smokers [6], and nearly half using cannabis regularly [7]. Although data on opioid use in schizophrenia is limited, it was found to be significantly higher than the general population [8]. Substance use and dependence is a significant complication for patients with schizophrenia and often impedes treatment options. For example, cigarette smoking has been shown to have a profound effect on the metabolism of many psychotropic drugs, with plasma concentrations of clozapine shown to be reduced in people who smoke cigarettes. [9–11]. First-generation and short acting oral antipsychotics have also been shown to increase substance use and cravings, with alcohol use in patients worsening over time [12, 13].

Despite the high heritability and prevalence of SUDs in schizophrenia, genome-wide association studies (GWAS) for SUDs lag those for other major psychiatric disorders. In comparison to GWAS mega-analyses like schizophrenia and major depressive disorder, there is still the need for larger cohorts for SUD phenotypes to boost discovery power, particularly for psychostimulants such as cocaine and amphetamines. Current GWAS on SUD phenotypes have not revealed any clear novel biological pathways involved in these phenotypes, with limited loci found in SUDs, thus more work in this area is needed. In terms of the genetic relationship between SUD phenotypes and schizophrenia, previous research has shown that the odds of substance use in individuals with schizophrenia is higher than the general population [6], Hartz et al. found that schizophrenia was significantly genetically correlated with nicotine dependence, cigarettes per day and ever/never smoking [14]. Furthermore, Reginsson leveraged data from Icelandic subjects (*N* = 144,609) to demonstrate the elevated SZ polygenic risk score was associated with increased risk of alcohol and substance use disorders [15]. The largest GWAS to date for life-time cannabis use was significantly genetically correlated with schizophrenia, with Mendelian randomisation analysis showing evidence for a causal positive influence of schizophrenia risk on cannabis use [16]. Despite the substantial amount of research supporting the shared genetic overlap of SUDs and SZ, the aetiology of SUDs in SZ still remains relatively uncharacterised. In the current study, we employed pairwise meta-analysis GWAS to reveal genes potentially underpinning substance dependence in schizophrenia, that may not be apparent through investigation of the disorders individually. Understanding the molecular determinants of comorbid addiction in schizophrenia has the potential to identify new therapeutic targets, which may lead to improved clinical outcomes in those individuals with risk of these exacerbating conditions.

## Materials and methods

### Genome-wide association studies (GWAS)

GWAS Summary statistics of European ancestry were obtained for schizophrenia (*N* = 130,644) and substance use and dependence from the Psychiatric Genomics Consortium (PGC), International Cannabis Consortium (ICC) and GWAS & Sequencing Consortium of Alcohol and Nicotine use (GSCAN). Substance use and dependence GWAS include Alcohol Dependence (unrelated genotyped individuals) with alcohol exposed controls (AD, *N*_cases_ = 8485*, N*_controls_ = 20,272) [17], Nicotine Dependence UKB2 (ND, *N*_cases_ = 10,287*, N*_controls_ = 234,603) [18], Life-time Cannabis Use excluding 23&me [16], (LCU, *N*_cases_ = *43,380, N*_controls_ = 118,702), Cannabis Use Disorder (unrelated genotyped individuals) [19] (CUD, *N*_cases_ = *1*4,080*, N*_controls_ = 343,726), Opioid Dependence (unexposed controls) [20] (OD, *N*_case*s*_ = 3272*, N*_controls_ = 25,437), which included individuals with no life-time OD diagnosis who were not exposed to illicit or prescription opioids as controls, Cigarettes per day [18, 21, 22] (CPD, *N* = 337,334), and Drinks per week [22] (DPW, *N* = 941,280). All AD, CUD, and OD cases met criteria for a life-time diagnosis of DSM-IV derived from clinician ratings or semi-structured interviews, ND had the ICD-10-CM Diagnosis Code F17 recorded from in their hospital records.

### Genetic correlation and evidence of causation

Linkage disequilibrium score regression analysis (LDSC, v 1.0.1) was utilised to estimate genetic correlation between schizophrenia and each SUD phenotype [23]. Common SNPs (MAF > 0.05) in the GWAS summary data were retained if they were available in the HapMap3 panel that excluded the MHC region and were not otherwise excluded by the ‘munge_sumstats.py’ script in the LDSC framework. Genetic correlation between two traits may be indicative of shared underlying biology but does not necessarily imply that the relationship is causal. To evaluate evidence for a causal relationship between SUD phenotypes and schizophrenia, we constructed a latent causal variable model [24] (LCV), as has been demonstrated elsewhere [24–28]. Briefly, the LCV model utilises the genome-wide SNP-trait association *Z*-scores for two traits and the mixed fourth moments (cokurtosis) of the respective distributions to assess whether there is evidence for a causal effect of one trait on the other. As a result, the LCV framework can calculate a metric termed the posterior mean causality proportion (GCP), from which the sign can be used to infer a potential causal direction. In practice, GCP > 0 implies that trait one is partially genetically causal for the second trait, while GCP < 0 implies the reverse. Given GCP > 0, for example, marginal SNP-trait one effect sizes tend to be proportionally larger on trait two, but this is not observed in the opposite direction—hence, the direction of causal effect can be estimated as operating from trait one to trait two. We defined partial genetic causality using the recommended threshold of a significantly non-zero |GCP | > 0.6, as this was shown by O’Connor and Price in simulations to guard against false positives [24]. It should be noted that the posterior mean GCP is not an estimate of the magnitude of any potential causal relationship and should not be interpreted as such—rather it evaluates the strength of evidence for a putative causal relationship in either direction using genome-wide SNP effects.

### Gene-based association analysis

Gene-based association was performed on each disorder using MAGMA version 1.07 (https://ctg.cncr.nl/software/magma). The MAGMA gene-based method utilises *P*-values as input, whereby the test-statistic is a linear combination of SNP-wise *P*-values. In comparison to univariate GWAS, the burden of multiple-testing correction is dramatically reduced in gene-based association analysis [29]. Gene-based association can also greatly boost power by signal aggregation across variants in the target regions when multiple causal variants influence the phenotype of interest [30]. The default gene-based test was used, a modified version of Brown’s method for combining *P*-values such that test-statistic inflation arising from SNP-wise dependency due to LD within genes can be suitably accounted for. The 1000 genomes phase 3 European reference panel is used as the LD reference for this purpose. Variants were mapped to 18,297 autosomal protein-coding genes from NCBI hg19 genome-assembly. Genes that arise from the major histocompatibility complex (MHC, chr6:28477797–33448354) on Chromosome 6 were removed. Statistical inference for a significantly associated gene for each disorder was set as *P* < 2.7 × 10^−6^ to adjust for the number of genes tested via the Bonferroni method.

### Pairwise cross-disorder meta-analysis

The genic *Z*-score outputs from the gene-based association analysis for schizophrenia and each substance dependence phenotype (AD, CUD, ND, OD), which were probit transformation of the *P*-values, were meta-analysed individually using MAGMA—that is, schizophrenia was meta-analysed with AD, then with CUD, and so on. A weighted *Z*-test was utilised for this purpose, which is based off the inverse normal (Stouffer’s) method, whereby the weight (*w*_i_) was set as the respective GWAS sample sizes [31]. Given the LD score regression (LDSR) intercept was markedly non-zero for AUD with schizophrenia in the LDSR model (*P* < 0.01), which may be driven by sample overlap, we compared our Stouffer’s method meta-analytic *P*-values for schizophrenia with AUD to those derived from the Cauchy combination test method [32]. In this method, the test-statistic is a sum of the schizophrenia and AUD *P*-values, per gene, transformed to approximate a Cauchy distribution, which is also flexible to incorporate weights of the input study sample sizes. The test-statistic is insensitive to correlations among the *P*-values that arise due to these *P*-values being from the same sample due to the heavy tail of the Cauchy distribution, with the combined meta-analytic *P*-value approximated using the cumulative density function of the Cauchy distribution. We calculated the Pearson’s correlation estimate, with 95% confidence intervals (CI), comparing the negative logarithm base ten transformed *P*-values of both methods.

### Gene-set association analyses

Competitive gene-set analysis results were obtained using MAGMA. For this analysis, 14,969 hallmark, 2921 canonical and 2598 regulatory miRNA target gene ontology gene-sets from the molecular signatures database (MsigDB, v7.4) were selected [33, 34]. A linear regression model is constructed by MAGMA wherein genic association (transformed to Z) is the outcome. Confounders that are adjusted for in this analysis include gene-size and genic-minor allele count. Gene-set association analysis was undertaken to find gene-sets where the common variant signal is enriched relative to all other genes considered. Genes that share biological or functional properties from a defined reference database are aggregated into sets that include molecular interactions, regulation, and products to determine pathways relevant to the phenotype of interest. Multiple-testing correction for the gene-set analysis was performed using the Benjamini–Hochberg (BH) procedure, with FDR < 0.05 designated as a significant gene-set association [35]. For functional enrichment analysis of microRNA target gene-sets, we used the web server g:Profiler [36].

## Results

### Evidence for partial genetic causality of alcohol dependence on schizophrenia

**Fig. 1:**
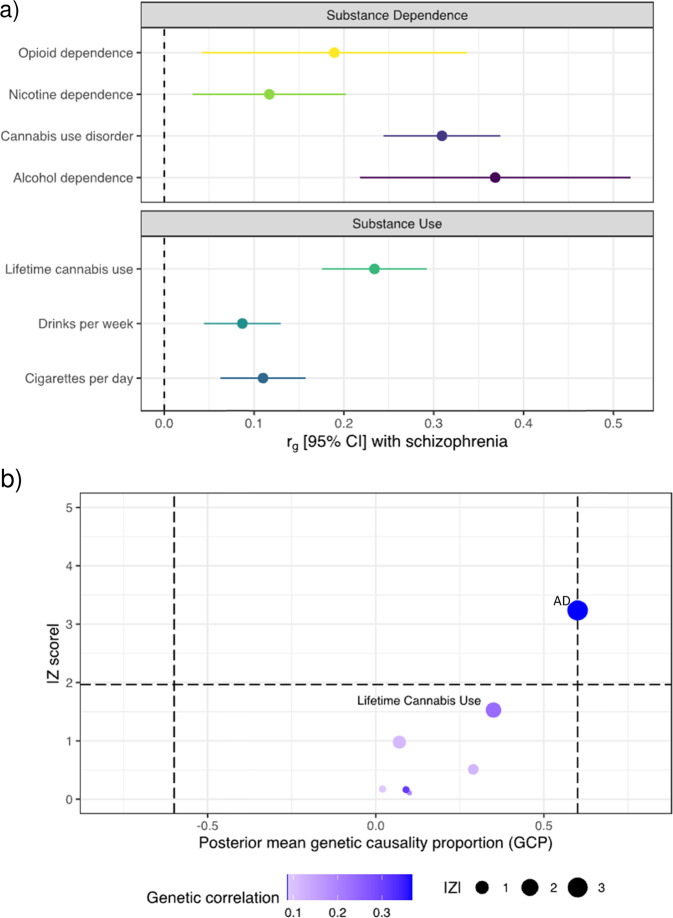
Genetic relatioships between substance use disorders and schizophrenia. **a** Genetic correlation forest plot between schizophrenia and SUDs (AD, CUD, ND, and OD), *r*_*g*_ calculated using linkage disequilibrium (LD) score regression. **b** Latent causal variable (LCV) model to test for partial causality with posterior mean genetic causality proportion (GCP) estimates of each SUD on schizophrenia. Absolute magnitude of the *Z*-score relates to the test of whether the posterior mean GCP estimate is significantly different than zero. The vertical line represents genetic causality, with a posterior mean GCP > 0.6 implying partial genetic causality of trait 1 and trait 2, and inverse for GCP < −0.6. The horizontal line represents an absolute *Z*-score of ~1.96, which is nominal uncorrected significance (*P* < 0.05) for a test of whether GCP value is significantly non-zero.

LD score regression (LDSR) analysis revealed significant genetic correlations between schizophrenia and several of the SUD phenotypes after correcting for the number of tests performed (Fig. 1a). Specifically, schizophrenia was positively genetically correlated with AD (r_*g*_ = 0.368, SE = 0.076, *P* = 1.61 × 10^−6^*)*, CUD (*r*_*g*_ = 0.309, SE = 0.033, *P* = 1.97 × 10^−20^), ND (*r*_*g*_ = 0.117, SE = 0.043, *P* = 7.0 × 10^−3^), and the substance use phenotypes CPD (*r*_*g*_ = 0.11, SE = 0.024, *P* = 4.93 × 10^−6^), DPW (*r*_*g*_ = 0.087, SE = 0.021, *P* = 6.36 × 10^−5^) and LCU (*r*_*g*_ = 0.234, SE = 0.029, *P* = 3.74 × 10^−15^). We also found nominally positive genetic correlation between schizophrenia and OD (*r*_g_ = 0.184, SE = 0.075, *P* = 0.0142), however, this did not survive multiple-testing correction. LCV models were then constructed for the significant traits for which their schizophrenia genetic correlation estimate passed multiple-testing correction (AD, CUD, ND, CPD, DPW, and LCU) to investigate whether any of the observed genetic correlations between SUD and schizophrenia may constitute a causal relationship (Fig. 1b, Supplementary Table 1). There was no evidence for partial genetic causality of CUD, ND, CPD, DPW, and LCU on schizophrenia, but there was moderate evidence that AD was partially genetically causal for schizophrenia (GCP = 0.60, SE = 0.22, *P* = 0.001). We note that while the SNP heritability estimate for AD was significantly non-zero, the *Z*-score for AD (*h*^2^/SE) was somewhat noisier (*Z*_*h*_^*2*^ = 5.98) than recommended by the authors of the LCV method (*Z*_*h*_^*2*^ > 7). As a result, this inference of partial genetic causality needs to be cautiously interpreted in light of this, with larger GWAS of AD diagnosed using DSM-IV/DSM-V or similar likely required to boost the precision of AD SNP heritability. DPW did not show any evidence for a causal relationship with schizophrenia like alcohol dependence. Interestingly, AD and DPW showed genetic correlation (*r*_*g*_ = 0.709, SE = 0.105, *P* = 1.38 × 10^−11^), as did CUD and LCU (*r*_*g*_ = 0.476, SE = 0.049, *P* = 2.71 × 10^−22^) but there was no evidence of partial genetic causality of DPW on AD (GCP = 0.05, SE = 0.56, *P* = 0.88) or CUD and LCU (GCP = 0.02, SE = 0202, *P* = 0.976). No evidence of genetic correlation was observed among ND and CPD (*r*_*g*_ = 0.071, SE = 0.054, *P* = 0.192). These results suggest that the underlying mechanisms driving AD, CUD, and ND may not strongly present in substance use observed in a population sample, although this requires further investigation.

### Gene-based multivariate association reveals 44 novel signals for substance dependence and schizophrenia

Using aggregated gene-level association (MAGMA) on the individual traits, we observed that 534 genes were associated with schizophrenia alone after Bonferroni correction (*P* < 2.7 × 10^−6^, Supplementary Table 2). For cannabis use disorder, 2 genes were significantly associated including, *PDE4B* (*P* = 2.09 × 10^−6^) and *FOXP2* (*P* = 9.30 × 10^−7^, Supplementary Table 3), and one gene for nicotine dependence—*ARHGAP22* (*P* = 2.42 × 10^−6^, Supplementary Table 4). While no genes were associated with AD and OD after multiple-testing correction, 9 genes were significantly associated with life-time cannabis use: *LRRTM4* (*P* = 4.51 × 10^−7^), *CADM2* (*P* = 1.14 × 10^−13^), *AS3MT* (*P* = 9.43 × 10^−7^), *NCAM1* (*P* = 1.44 × 10^−9^), *ATXN2L* (*P* = 1.85 × 10^−8^), *TUFM* (*P* = 5.72 × 10^−8^), *SH2B1* (*P* = 3.15 × 10^−8^), *ATP2A1* (*P* = 1.68 × 10^−8^), *RABEP2* (*P* = 1.64 × 10^−6^), and *SRR* (*P* = 1.61 × 10^−6^) (Supplementary Table 5) with none of these genes shared with CUD.

**Fig. 2:**
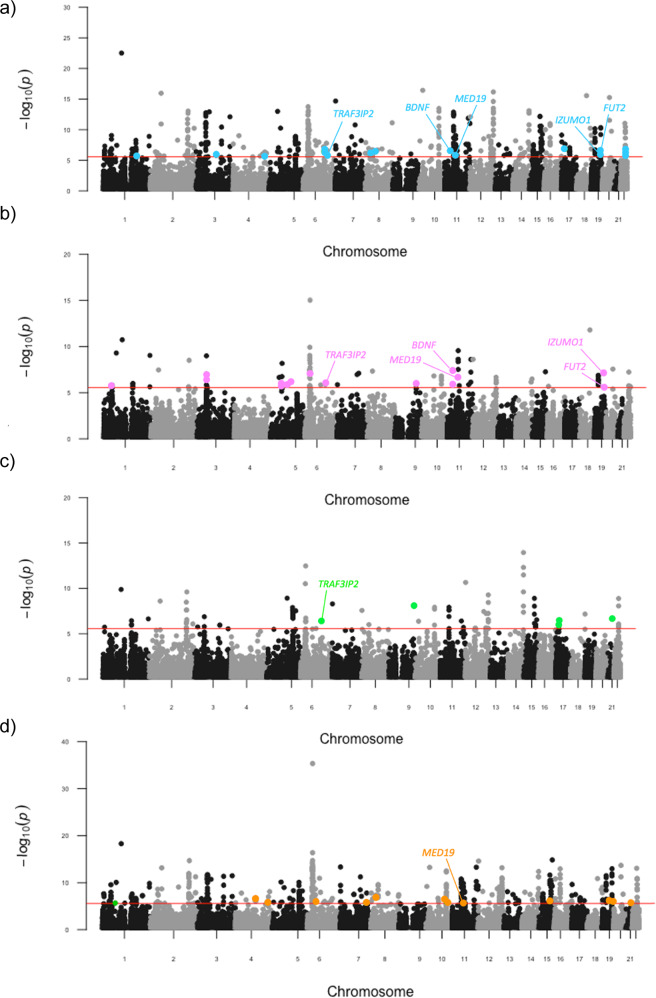
Pairwise genic meta-analyses of schizophrenia and SUDs. Manhattan plot for each meta-analysis that displays the −log10-transformed *P*-value for association for genes, which were tagged by at least one SNP in the respective GWAS. The red line represents the Bonferroni threshold for multiple-testing correction (*P* < 2.7 × 10^–6^). Genes highlighted on each plot were not Bonferroni significant in the individual GWAS, with overlapping genes across meta-analyses labelled. **a** SZ and AD, **b** SZ and CUD **c** SZ and ND, **d** SZ and OD.

The genetic architecture shared between schizophrenia and substance dependence was further investigated using genic pairwise meta-analysis (MAGMA) to identify potentially pleiotropic signals that do not reach conventional significance thresholds in the individual GWAS (Fig. 2). The number of genes that survived Bonferroni correction in each of the meta-analyses was as follows: 444 genes for schizophrenia meta-analysed with OD, 437 genes for schizophrenia meta-analysed with AD, 94 genes for schizophrenia meta-analysed with CUD, and 99 for schizophrenia meta-analysed with ND. The top hit for schizophrenia, *HIST1H4* (*P* = 2.86 × 10^−38^), was also the most significant for the schizophrenia meta-analysis with AD (*P* = 2.70 × 10^−31^), and OD (*P* = 4.50 × 10^−36^). Whereas the most significant gene observed in the CUD and schizophrenia meta-analysis was *ST3GAL3* (*P* = 9.68 × 10^−16^) and in the ND and schizophrenia meta-analysis the gene *ZFYVE21* (*P* = 1.11 × 10^−14^). We then restricted these genes to those which were also at least nominally significant (*P* < 0.05) in the individual GWAS for schizophrenia and SUD but did not survive multiple-testing correction for either of the respective univariable GWAS (schizophrenia + AD = 16, schizophrenia + CUD = 15, schizophrenia + ND = 5, schizophrenia + OD = 13, Supplementary Tables 6–9). These genes are likely more biological salient, as many of the other, which survived correction in the meta-analyses that did not reach nominal significance in the respective SUD GWAS were driven purely by schizophrenia given its greater discovery power. Interestingly, five of these genes were found in more than one bivariate meta-analysis; specifically, (1) *TRAF3IP2* and (2) *MED19* in the schizophrenia + AD, schizophrenia + CUD, and schizophrenia + ND meta-analyses; along with (3) a *BDNF*, (4) *FUT2* and (5) *IZUMO1* for both the schizophrenia + AD and schizophrenia + CUD meta-analysis (Table 1). In the schizophrenia with AUD meta-analysis, which demonstrated evidence of sample overlap due to the non-zero LDSR intercept, we found that leveraging the Cauchy distribution to ensure the meta-analytic *P*-values was not inflated by sample overlap yielded highly similar results to the default Stouffer’s method (*r* = 0.9744 [95% CI: 0.9737, 0.9752]).

**Table 1:** The top ten significantly associated genes observed in the schizophrenia and substance dependence (AD, CUD, ND, and OD) MAGMA meta-analyses that were nominally significant (*P* < 0.05) in the individual GWAS but did not pass multiple-testing corrections.

Gene symbol	*P*_SZ_	*P*_SUD_	*P*_META_
*MED27*	6.47 × 10^−5^	ND, 1.425 × 10^−5^	SZ + ND, 8.64 × 10^−9^
*BDNF*	4.22 × 10^−6^	AD, 1.08 × 10^−2^	SZ + AD, 2.75 × 10^−7^
		CUD, 1.39 × 10^−4^	SZ + CUD, 3.42 × 10^−8^
*IZUMO1*	2.29 × 10^−5^	AD, 6.52 × 10^−3^	SZ + AD, 1.05 × 10^−6^
		CUD, 1.13 × 10^−4^	SZ + CUD, 7.67 × 10^−8^
*HYAL3*	2.06 × 10^−5^	CUD, 1.47 × 10^−4^	SZ + CUD, 9.83 × 10^−8^
*HMGN4*	3.65 × 10^−4^	CUD, 2.63 × 10^−5^	SZ + CUD, 1.00 × 10^−7^
*EP300*	1.18 × 10^−5^	AD, 9.72 × 10^−4^	SZ + AD, 1.36 × 10^−7^
*SPECC1*	8.28 × 10^−6^	AD, 1.99 × 10^−3^	SZ + AD, 1.51 × 10^−7^
*PPP2R2A*	2.95 × 10^−6^	OD, 9.82 × 10^−3^	SZ + OD, 1.78 × 10^−7^
*MED19*	1.16 × 10^−5^	AD, 2.82 × 10^−2^	SZ + AD, 1.74 × 10^−6^
		CUD, 3.53 × 10^−4^	SZ + CUD, 2.01 × 10^−7^
		OD, 3.25 × 10^−2^	SZ + OD,, 1.99 × 10^−6^
*FUT2*	3.37 × 10^−6^	AD, 1.02 × 10^−2^	SZ + AD, 2.10 × 10^−7^
		CUD, 4.18 × 10^−3^	SZ + CUD, 2.55 × 10^−6^

### Bivariate gene-set association meta-analysis uncovers novel biological systems involved in schizophrenia and substance use disorders

We investigated the involvement of 14,969 gene-sets (MsigDB v7.4) in four categories including Biological Process (BP), Cellular Component (CC), Molecular Function (MF), and Human Phenotype Ontology (HPO), respectively. For the individual schizophrenia GWAS, 84 gene-sets remained statistically significant after multiple-testing correction (FDR < 0.05). No gene-sets passed false discovery rate (FDR) correction for the individual substance dependence phenotypes.

In the pairwise meta-analysis, 73 gene-sets were statistically significant (FDR < 0.05), for schizophrenia meta-analysed with AD, with 10 gene-sets not previously observed in the individual schizophrenia GWAS (Supplementary Table 10), including *long-term synaptic potentiation* (*n*_genes_ = 81, *P* = 5.71 × 10^−5^, FDR = 0.02), *exocytic vesicle* (*n*_genes_ = 196, *P* = 1.23 × 10^−4^, *FDR* = 0.03), *paroxysmal ventricular tachycardia* (*n*_genes_ = 23, *P* = 1.70 × 10^−4^, *FDR* = 0.03), *peptidyl serine dephosphorylation* (*n*_genes_ = 19, *P* = 1.75 × 10^−4^, *FDR* = 0.04), *hypoplasia of the olfactory bulb* (*n*_genes_ = 4, *P* = 2.12 × 10^−04^, *FDR* = 0.04), *regulation of heart contraction* (*n*_genes_ = 221, *P* = 2.32 × 10^−4^, FDR = 0.04), and *regulation of peptidyl serine dephosphorylation* (*n*_genes_ = 5, *P* = 2.32 × 10^−4^, FDR = 0.04). The schizophrenia and CUD meta-analysis yielded 33 statistically significant sets, with 12 not observed in the individual SZ GWAS (Supplementary Table 11). Some of these novel gene-sets of interest were; *abnormal social behaviour* (*n*_genes_ = 140, *P* = 7.46 × 10^−7^, FDR = 0.002), *aggressive behaviour* (*n*_genes_ = 165, *P* = 1.75 × 10^−5^, FDR = 0.018), *hypoplasia of the olfactory bulb* (*n*_genes_ = 4, *P* = 7.24 × 10^−6^, *FDR* = 0.01), and *obsessive compulsive behaviour* (*n*_genes_ = 90, *P* = 6.66 × 10^−5^, *FDR* = 0.04). There were 60 gene-sets passed FDR correction in the schizophrenia meta-analysis with OD (Supplementary Table 12), with 9 not previously seen in individual SZ GWAS, including *spherical high-density lipoprotein particle* (*n*_genes_ = 8, *P* = 6.84 × 10^−5^, *FDR* = 0.02), *histone deacetylase complex* (*n*_genes_ = 70, *P* = 9.25 × 10^−5^, FDR = 0.03), and *aggressive behaviour* (n_genes_ = 165, *P* = 1.68 × 10^−5^, FDR = 0.04). Finally, schizophrenia meta-analysed with nicotine dependence yielded the fewest significant gene-sets (31 gene-sets, Supplementary Table 13), however, 10 sets were still novel relative to what was observed in each individual GWAS, such as: *neurotrophin receptor binding* (*n*_genes_ = 11, *P* = 2.85 × 10^−5^, FDR = 0.02), *glutamatergic synapse* (*n*_genes_ = 284, *P* = 4.00 × 10^−5^, FDR = 0.03), *long-term synaptic potentiation* (*n*_genes_ = 81, *P* = 4.69 × 10^−05^, FDR = 0.03), and *type I pneumocyte differentiation* (*n*_genes_ = 5, *P* = 7.70 × 10^−5^, FDR = 0.03). Interestingly, the human behaviour ontology set *abnormal aggressive impulsive* or *violent behaviour* and *aggressive behaviour* was common among schizophrenia meta-analysed with CUD, ND or OD, while *long-term synaptic potentiation* (*LTP*) was also common between schizophrenia and AD and ND.

We then considered the association of 2,598 microRNA (miRNA) regulatory target prediction gene-sets with each individual GWAS, followed by the bivariate meta-analyses (Supplementary Tables 14–18). There were 239 miRNA that passed FDR correction for the individual schizophrenia GWAS, no miRNA regulator target gene-sets passed FDR correction for any of the individual substance dependence phenotypes. Notably, each of the meta-analyses revealed a total of 17 microRNA regulatory target gene-sets, not seen in the individual phenotypes, including six found in more than one bivariate meta-analysis. One such interesting example was the predicted target genes of miR-495, a microRNA that is highly enriched in the nucleus accumbens and has been shown to play a role in addiction-related behaviours [37]. MiR-495 survived correction in both schizophrenia and AD meta-analysis (*n*_genes_ = 231, *P* = 2.43 × 10^−3^, FDR = 0.03), and the schizophrenia and ND model (*n*_genes_ = 231, *P* = 5.88 × 10^−5^, FDR = 0.01) but was only nominally significant in the individual schizophrenia GWAS (*P* = 0.007), supporting how this meta-analysis approach can increase discovery power.

The miRNA miR-137 is one of the most well studied genes implicated by schizophrenia GWAS, the regulatory targets of miR-137 (*n*_genes_ = 487) were significantly enriched with schizophrenia associated variation considering the individual schizophrenia GWAS (*P* = 4.54 × 10^−5^, FDR = 0.004). However, meta-analysis of schizophrenia with each of the substance dependence phenotypes did not increase this association, suggesting the signal was more localised to schizophrenia upon considering the currently available GWAS. We hypothesised that the biology associated with targets of miR-137 may be shared with that of the 17 novel miRNA target sets implicated by the meta-analysis. To test this, we evaluated which biological pathways and other ontological gene-sets the targets of miR-137 were overrepresented in relative to the 17 novel miRNA target sets uncovered. We found that there were several neuronal related processes enriched among the targets of miR-137 and at least one of the 17 miRNAs’, such as *neurogenesis, nervous system development*, and *glutamatergic synapse* and several implicated in metabolic processes.

## Discussion

In this study we used genetic approaches to explore common genes between schizophrenia and substance dependence using GWAS that met strict criteria for a life-time diagnoses. As most available substance use and misuse GWAS only measure a recent state instead of a stable trait, and substance dependence is likely neurodevelopmental in origin, most current SUD GWAS likely do not capture life-time substance dependence. A 2020 study by Zhou et al. on problematic alcohol use (PAU) [38] and a 2021 study by Johnson et al. [39] on alcohol use disorder (AUD) also investigated the genetic overlap with schizophrenia, using the Million Veterans Program (MVP) Phase 1 summary statistics reliant on Alcohol Use Disorders Identification Test (AUDIT-C), and the UK biobank (UKBB) summary statistics that use AUDIT-P. Although Zhou et al. showed genetic correlation among the entire MVP AUD and PGC AD (*r*_*g*_ = 0.98, SEM = 0.11, *P* = 1.99 × 10^−19^), the AUDIT is still limited to the past 12-months [40, 41]. Though the PGC summary statistics are smaller in sample size, they offer a more precise assessment of alcohol dependence [17]. Further work is required to refine phenotypes that index dependence and balance available sample sizes verses the informativeness of the metric.

In this study, we further supported previously known evidence of genetic correlation between schizophrenia and these substance dependence phenotypes, while there was further evidence of a causal relationship between alcohol dependence and schizophrenia. Interestingly, there was no causal relationship between the consumption of alcohol (drinks per week) and schizophrenia, or between AD and DPW. This was consistent with the trans-ancestral GWAS of alcohol dependence [17], which suggested there is a distinction in the underlying molecular mechanisms driving pathological and non-pathological behaviours for substance use and dependence, particularly within biological pathways implicated in the psychopathological aspects of problematic drinking [42]. Additionally, it is also well known that psychotic symptoms can occur in several clinical conditions related to alcohol such as intoxication, withdrawal, alcohol-induced psychotic disorder, and delirium [43]. Although registry data-sets come with several limitations such as the threat of false-negatives due to under-reporting of substance use, a study on 18,478 Finnish inpatients found alcohol-induced psychosis was the most common type of substance-induced psychotic disorder (SIPD) [44], with a separate Swedish study that followed 7606 individuals for 84 months between 1995 and 2015 found that for alcohol the risk for SIPD was 4.7% [45]. Interestingly, 22.1% (95% CI = 17.6−27.5) of patients who had previously received a diagnosis of alcohol-induced psychosis went on to develop schizophrenia [46]. The putative causal relationship of AD on schizophrenia warrants further epidemiological and biological interrogation. There are also some important limitations to the use of LCV models—specifically, they are bivariate in nature, and thus, cannot model the effect of other plausible mediators or confounders, while the posterior mean GCP estimate is also not a causal estimate that could be afforded by approaches like Mendelian randomisation [24]. However, the use of Mendelian randomisation with a binary exposure like AD can be challenging [47], particularly as only a handful of genome-wide significant SNPs have been identified that could be suitable instrumental variables.

Strikingly, we also observed 44 genes associated with substance dependence in schizophrenia that were not seen in the individual GWAS. Five of these genes (*TRAF3IP2, MED19, BDNF, FUT2*, and *IZUMO1)* were common hits in more than one of the paired disorders, with the 3’ untranslated region (UTR) of *FUT2* previously reported by Jang et al. to be associated with continuous alcohol and psychiatric disorder phenotypes [48], and a cross-disorder analysis of pleiotropic SNPs on AUD and SZ by Johnson et al. that identified 55 convergent loci also reported *FUT2* as a nearby gene to one of these loci [39]. *TRAF3IP2*, encodes nuclear factor-kappa-B (NF-κB) activator 1 (Act1), an IL-17 receptor adaptor protein [49]. *TRAF3IP2* plays a critical role in the activation of multiple pro-inflammatory signalling pathways [50], particularly IL-17. IL-17 is a negative regulator of adult hippocampal neurogenesis, with the absence of IL-17 shown to significantly improve neurogenesis and enhance synaptic function [51]. Prenatal IL-17 expression has been shown to influence neurodevelopment because of its role in cell differentiation, signalling, and survival [51]. Mediator complex subunit 19 (*MED19*) is a physical and functional target of RE1 silencing transcription factor (REST), with combined depletion of MED19/MED26 shown to result in de-repression of REST targets in vivo [52]. REST is the master transcription factor of neuron-specific genes, particularly during postnatal brain development [53], and has been shown to modulate μ-opioid receptor (MOR) gene expression [54]. The Mu opioid receptor (MOR) is a key modulator of the dopaminergic system, with the rewarding properties of opioids and non-opioid drugs shown in Oprm1−/− mice to be reduced or eliminated [55].

*MED19* is also a mediator of peroxisome proliferator-activated receptor gamma (PPARγ) transcriptional activity [56], which is essential for adipogenesis and glucose uptake and storage [57]. Interestingly, in the schizophrenia and OD gene-set analysis, the gene-set *spherical high-density lipoprotein particle* was enriched. In humans, activation of PPARγ is generally associated with an increase in plasma HDL-cholesterol [58]. Adipose tissue is an endocrine gland, which secretes leptin and adiponectin, cytokines with pro- and anti-inflammatory properties, respectively [59]. The dysregulation of adipokine levels has been associated with schizophrenia and other related disorders [60], with Olanzapine, clozapine, and quetiapine shown to elevate the pro-inflammatory cytokine, leptin [61]. Cocaine- and amphetamine-regulated transcript (CART) production and action is also modulated by leptin [62]. Moreover, there is evidence that schizophrenia genetic risk among insulin and glycaemic related pathways could be a target of therapeutic intervention [63, 64].

Brain-derived neurotrophic factor (*BDNF*) has long been linked to neurodegenerative diseases and psychiatric disorders such as substance dependence [65]. *BDNF* plays an important role in the regulation of synaptic strength and plasticity in the brain [66], glucose metabolism and the regulation of mammalian food intake via signalling in hypothalamic circuits [67, 68]. Hyperphagic obesity has been shown to develop in humans heterozygous for *BDNF* [69, 70]. Notably, we also detected a novel, Bonferroni significant association with *BDNF* upon meta-analysis of schizophrenia with alcohol dependence and cannabis use disorder. In the gene-set analyses, *neurotrophin receptor binding* was also a novel ontological pathway significant for schizophrenia and ND. Additionally, several of the novel 44 genes have also been found to play important roles in metabolism. In mouse studies, protein tyrosine kinase 2 beta (PTK2B) was found to play a critical role in the differentiation of beige adipocytes [71], CD47–/– knockout resulted in resistance to insulin desensitisation, glucose intolerance and diet-associated weight gain [72]. *Spherical high-density lipoprotein particle* was also a significant pathway in the Schizophrenia and OD meta-analysis.

The ontologies enriched for the 17 microRNA regulatory target gene-sets not seen in the individual phenotypes, that were significant in the schizophrenia and substance dependence analyses also revealed some biologically salient insights. For instance, miR-5580 target genes were enriched in *insulin receptor signalling pathway* and miR-7856 target genes were enriched in several pathways implicated in the regulation of glucose metabolism. miR-137 target gene-sets were also enriched in several metabolic pathways including r*egulation of cellular response to insulin stimulus*. Literature has shown that microRNAs may regulate gene networks involved in disorders like schizophrenia [73]. miR-495 also directly targets the 3’ UTR of BDNF, with overexpression of miR-495 found to suppress cocaine self-administration in mice [37].

This study explores the molecular determinants of shared vulnerability in SUDs and SZ, however, this work has several limitations. Firstly, the binary SUD phenotypes used different diagnostic criteria; specifically, AD, CUD, and OD used the DSM-IV and DSM-V, while ND was derived from inpatient ICD-10 codes. Second, as there is significant heterogeneity in the cases and controls, it is difficult to determine if the phenotypes in the samples studied were using other substances or had a history of dependence to other substances. As a result, significant enrichment of genes and gene pathways for the shared SZ and SUD genetic signals may arise due to either a comorbidity and/or pleiotropy with other dependence phenotypes and do not imply causal biology that would necessarily be beneficial to either schizophrenia and the substance use disorder phenotypes. The studies where case numbers are smaller could also result in an inflated effect size of associated variants, and our shared genes may be the result of being in linkage disequilibrium with the causal gene. The difference between exposed and unexposed controls is also a potential confounder, particularly for the OD GWAS, as OD only requires exposed controls have a history of opioid use (prescription and/or illicit), it does not account for potential behavioural differences in subjects who have been exposed to prescription opioids as compared to those which are illicit. Future GWAS of polysubstance dependence would also be invaluable to disentangle some of the complex biological drivers of these disorders. Lastly, the findings in this study are limited to European/caucasion populations only, and give an incomplete picture of the genetic underpinnings of SUDs comorbid with schizophrenia.

Despite the significant disease comorbidity in schizophrenia and substance dependence, previous GWAS for these disorders failed to reveal large overlap between genome-wide significant hits. We demonstrate that the increase in power afforded through our pairwise meta-analysis approach was able to identify shared genetic signals, including new genes and biological pathways relevant to both the neurobiology of addiction and psychosis. These novel genes and biological systems should be used in future analyses to refine whether causal variation is mapped to these genes, and whether any of these signals could warrant therapeutic intervention.

## Supplementary information

Related Manuscript File
